# Simultaneous diagnosis of severe imported *Plasmodium falciparum* malaria and HIV: report of three cases

**DOI:** 10.1186/s12936-015-0780-6

**Published:** 2015-07-10

**Authors:** Nuno Rocha Pereira, António Sarmento, Lurdes Santos

**Affiliations:** Infectious Diseases Department of Sao Joao Hospital Centre, Faculty of Medicine of University of Porto, Alameda Prof. Hernâni Monteiro, 4200 Porto, Portugal; Nephrology Research and Development Unit, Instituto de Engenharia Biomédica, I3S, Instituto de Investigação e Inovação em Saúde, University of Porto, 4200-319 Porto, Portugal

**Keywords:** Malaria, HIV, Imported malaria, Severe malaria, *Plasmodium falciparum*

## Abstract

The increasing number of travellers to and from areas where considerable overlap between high malaria transmission and elevated prevalence of human immunodeficiency virus (HIV) infection exists, augment the probability that returning travellers to non-endemic countries might present with both infections. The presence of such co-infection can increase the severity of malaria episodes and also can change the progression of HIV infection. This article describes three travellers returning from malaria-endemic areas that had simultaneous diagnosis of severe *Plasmodium falciparum* malaria and HIV infection. Despite the severe forms of malaria and HIV co-infection, all patients responded successfully to anti-malarial treatment. Malaria and HIV interact with one another, with HIV infection increasing parasite burden, clinical severity and risk of complications of malaria; malaria seems to create an immunological interaction favourable to HIV spread and replication, with impact in progression to AIDS. The presence of malaria and HIV co-infection also poses other challenges related to treatment response, level of care and possible interactions of drugs. The authors recommend that all patients with fever returning from malaria endemic areas should be screened both for malaria and HIV infection.

## Background

Every year about 25–30 million individuals travel from Europe to areas with malaria transmission [1] and around 25,000 cases of malaria occur, most of them having an uncomplicated course [2]. However rare, cases of severe imported malaria do occur and constitute a challenge for caregivers in non-endemic countries. Considerable overlap exist in geographical distribution of malaria and human immunodeficiency virus (HIV) infections [3] and the interaction between the two diseases raises new questions in the management of such patients, both in endemic and non-endemic countries. While the association between HIV infection and more severe clinical forms of malaria is well established in endemic countries [4–6], in non-endemic countries the importance of this interaction is largely unknown.

In particular, Portugal maintains firm relations with Portuguese-speaking African countries that are not only one of the most common destinations of Portuguese travellers and migrants but also the most common countries of origin of migrants entering in Portugal. These countries, namely Angola and Mozambique, are representative of the previously mentioned geographic overlap of malaria transmission and high prevalence of HIV infection, so with increasing numbers of people travelling to and from this destinations and with longer periods of stay, it is expected that some cases of severe malaria and HIV infection can be diagnosed simultaneously. Giving the considerable overlap of symptoms of both diseases in acute phase, high index of suspicion will be necessary to diagnose both conditions. The authors report three cases of severe imported *Plasmodium falciparum* malaria and HIV infection diagnosed simultaneously.

## Case presentations

### Case report 1

A 24-year-old woman from Angola presented at a University tertiary care hospital in Porto with a three-day history of fever, nausea, vomiting and diarrhoea. She had travelled from Angola, where she lived her entire life, to Portugal 9 days before. Her past medical history was unremarkable. On examination, she was febrile (38.4°C) and tachycardic (112 bpm), blood pressure and peripheral oxygen saturation were both normal. Laboratory data revealed leucocytosis (12.48 × 10^9^/L), slightly increased C-reactive protein (7.2 mg/dL), haemoglobin within normal range, normal platelet count, unaltered creatinine and normal transaminases and bilirubin. A thin blood film revealed the presence of *P. falciparum* with 4% parasitaemia. A 4th generation HIV test was also performed and had a doubtful result. Despite absence of severity criteria, the patient was admitted to infectious diseases (ID) ward for surveillance, treatment and further investigation of a probable HIV infection. She was started on oral quinine and doxycycline. In the following day, the patient presented altered mental status and blurred speech, without focal neurological deficits. A brain CT scan showed diffuse cerebral oedema and the thin blood film revealed a reduction in parasitaemia to 0.3%. Repeated 4th generation HIV test was positive. She was admitted to infectious diseases intensive care unit (ID ICU) and initiated intravenous treatment for malaria with quinine and clindamycin and anti-oedema therapy with mannitol. During her stay in ID ICU thrombocytopenia, altered coagulation tests and hyperbilirubinemia appeared. Clinical evolution was favourable and the patient was transferred to ID ward 4 days after admission in ID ICU. Complimentary study confirmed HIV infection with a viral load of 433,000 copies/mL and a T CD4 + lymphocyte count of 550 cells/mm^3^. She was discharged 12 days after admission and was referred to HIV clinic for follow-up (Table 1).

**Table 1:** Patients’ characteristics

	Patient 1	Patient 2	Patient 3
Age (years)	24	41	47
Sex	Female	Male	Male
Length of ICU stay (days)	4	37	5
Country of Malaria transmission	Angola	Angola	Mozambique
*P. falciparum* parasitaemia	4%	54%	1%
HIV viral load (copies/mL)	433,000 copies/mL	855,084 copies/mL	199,802 copies/mL
T CD4 + lymphocytes count/mm^3^	550	196	302

### Case report 2

A 41-year-old man from Portugal presented to the emergency room of a hospital with a 2-day history of fever and headache. He had been working in Angola in the last 3 years and had returned to Portugal 3 weeks before the beginning of symptoms. He reported a previous episode of malaria diagnosed in Angola treated with artemisinin derivatives. On examination he was febrile (39°C) and jaundice was apparent. Laboratory data revealed normal haemoglobin, thrombocytopenia (21 × 10^9^ platelets/L), leukopenia (2.8 × 10^9^/L) and elevated bilirubin (4.7 mg/dL). A thin blood smear revealed the presence of *Plasmodium,* but parasitaemia and species identification were not done. He was transferred to a University tertiary care hospital and was admitted to ID ICU. A repeated thin blood film showed a *P. falciparum* parasitaemia of 54%. A rapid diagnostic test was also performed and was positive for *P. falciparum* infection. Intravenous therapy with quinine and doxycycline was promptly initiated. On his 5th day at ID ICU and despite steady decrease in parasitaemia, the patient developed acute respiratory failure and shock, needing norepinephrine support and invasive mechanical ventilation. In the following day, the result of a 4th generation HIV test performed earlier was available and was positive. During his stay in ID ICU the patient also had avascular femoral head necrosis and a coagulase-negative *Staphylococcus* bacteraemia. Complimentary workup for HIV infection revealed a viral load of 855,084 copies/mL and T CD4 + lymphocyte count of 196 cells/mm^3^. After prolonged and difficult ventilator weaning, the patient was transferred to ID ward at his 37th day in hospital.

In the ID ward, the evolution was favourable and a repeated T CD4 + lymphocyte count performed after acute infections resolved revealed 188 cells/mm^3^. He started opportunistic infection prophylaxis with cotrimoxazole and after 44 days in the hospital, antiretroviral therapy with efavirenz and tenofovir/emtricitabine was initiated. He was discharged 47 days after admission and was referred to HIV and Travel Medicine clinics because he had the intention to return to Angola.

### Case report 3

A 47-year-old man from Portugal presented to the emergency room of a hospital in the same day of his return from Mozambique, where he had been working during the previous year, with diarrhoea, vomiting and abdominal pain with less than 24 h of evolution. He was prescribed domperidone and paracetamol and was discharged home. The symptoms persisted and a week later he also noticed the appearance of fever, jaundice and apathy. He went to the emergency room of another hospital 9 days after return. On examination jaundice and dehydration were apparent and he had normal heart rate and blood pressure. During is stay in emergency room he had a generalized tonic–clonic seizure. Laboratory data revealed anemia (9.9 g/dL), thrombocytopenia (17 × 10^9^ platelets/L), elevated bilirubin (10.0 mg/dL), elevated serum creatinine (7.76 mg/dL) and hyponatremia (127 mEq/L). A thin blood smear revealed the presence of *Plasmodium,* but species identification and parasitaemia were not done. He was transferred to a University tertiary care hospital and admitted to ID ICU. A repeated thin blood film revealed a *P. falciparum* parasitaemia of 1%. Intravenous therapy with quinine and doxycycline was initiated. At admission a 4th generation HIV test was also positive. He needed continuous venovenous haemodiafiltration during 4 days and then he was put on intermittent haemodialysis. At the 4th day in ICU parasitaemia was negative and in 5th day after admission quinine and doxycycline were switched to oral route and the patient was discharged to ID ward. In the ward he had good evolution and progressive recovery of kidney function was noticed. Complimentary workup for HIV infection revealed a viral load of 199,802 copies/mL and T CD4 + lymphocyte count of 302 cells/mm^3^. He was discharged home 10 days after admission and was referred to HIV clinic.

## Discussion

These three different patients illustrate several aspects of the malaria-HIV interaction. These two infections interact bidirectionally and synergistically with each other, so HIV can increase the severity of malaria, risk of adverse events and parasite burden while malaria can potentially accelerate HIV disease progression. All of the patients, without previously known HIV infection, experienced severe forms of *P. falciparum* malaria. Several reports from endemic countries show an increased prevalence and severity of clinical malaria in HIV infected patients [4, 6, 7]. However, these effects seem to depend from age, degree of immunosuppression and previous immunity to malaria [6]. Indeed, studies from endemic countries show increased prevalence of parasitaemia, clinical malaria and higher parasite density, increasing with the severity of immunosuppression, in adults with naturally acquired immunity to malaria [4, 6, 8]. Although the described interaction is extensively documented in endemic countries, in non-endemic countries data available about imported malaria and HIV co-infection is scarce. However, a study from Mouala et al. [9] in France revealed that, whatever the severity criteria used for malaria, HIV-infected patients with immunosuppression were at significantly higher risk of developing severe malaria than the non-HIV infected patients.

In case reports 1 and 3, the authors describe two patients with severe forms of malaria and HIV infection without advanced immunosuppression. The interaction between these two infections may explain to some extent malaria severity even in a previous immune adult (patient 1) and despite the absence of advanced immunosuppression. Conversely, in case report 2 the patient had some degree of malaria immunity but immunosuppression was present. HIV-infected patients with immunosuppression may present with higher parasite burden [6, 10] and this might explain the unusually high parasitaemia found in patient 2. Indeed the control of malaria parasitaemia is immune mediated and the immune deficiency caused by HIV infection can reduce the immune response to malaria parasitaemia and, therefore, increased parasite burden and frequency of clinical attacks of malaria are expected [8, 10]. Despite immunosuppression and very high parasitaemia, this patient had a favourable outcome probably as result of a relatively rapid initial diagnosis, early treatment and high-level supportive care provided in ICU. The importance of supportive care was describe in a report by Cohen et al. [11] that analysed patients treated in a tertiary care setting with ICU and dialysis. All three patients had relatively high HIV viral load, which can be related with malaria co-infection because malaria is associated with a strong CD4 + lymphocyte activation and up-regulation of pro-inflammatory cytokines, providing an ideal microenvironment for the spread of HIV among CD4 + cells and thus for a rapid replication that clinically can be seen by the increased HIV viral loads. All patients responded well to malaria treatment, despite that in Portugal the intravenous forms of artemisinin derivatives are unavailable. However, there is evidence of a possible impaired response to anti-malarials in HIV-patients, particularly those with immunosuppression [6].

Patient 2 intended to return to Angola as soon as possible. Clinical malaria is more frequent in HIV-infected patients, so the probability of repeat episodes of clinical malaria is high and this raises the question about potential interactions between antiretroviral therapy and anti-malarials [10]. Pharmacological interactions between these two classes of drugs are well documented in vitro, with the majority of them involving the cytochrome P450 [5]. Nonetheless, the importance of these interactions in clinical grounds is uncertain and specific guidelines for treatment of malaria in HIV-infected patients under antiretroviral treatment are lacking. Patient 2 initiated therapy with efavirenz and tenofovir/emtricitabine, a choice that may raise some important questions in a patient willing to return to an endemic area. According to World Health Organization recommendations, first line treatment for malaria in endemic areas should include an artemisinin derivative combined with lumefantrine, amodiaquine, sulfadoxine-pyrimethamine or piperaquine. Some reports document the potential interaction of efavirenz with a combination of artesunate and amodiaquine, resulting in increased transaminases and amodiaquine concentrations [6]. These reports led some authors to contraindicate amodiaquine containing regimens in patients under efavirenz [6]. The patient was also under prophylaxis with cotrimoxazole, which is an effective measure in reducing clinical malaria in HIV-infected patients but may raise concerns about development of resistance to antifolate anti-malarial drugs [4, 12].

Considerable overlap exists between symptoms of malaria, acute HIV infection and other opportunistic infections related to HIV, with the cardinal symptom of all those situations being fever. The traveller or migrant presenting in a non-endemic country with fever represents a clinical challenge and it is important that all clinicians recognise that malaria and HIV may co-exist and influence the clinical course of each other. Also early recognition of HIV co-infection in patients with malaria is important because the possible higher severity warrants more intense monitoring and a lower threshold for additional investigations to diagnose possible concomitant infections [13].

Considering the continuous movement of persons to and from endemic areas of malaria and with high prevalence of HIV, the authors recommend that all patients with fever returning from malaria endemic areas should be screened both for malaria and HIV infection.

### Consent

Written informed consent was obtained from the patients for publication of this Case report. A copy of the written consent is available for review by the Editor-in-Chief of this journal.

## Abbreviations

HIV: human immunodeficiency virus
ID: infectious diseases
ID ICU: infectious diseases intensive care unit
